# Effects of combined nitrogen and phosphorus application on soil phosphorus fractions in alfalfa (*Medicago sativa* L.) production in China

**DOI:** 10.3389/fpls.2024.1380738

**Published:** 2024-05-28

**Authors:** Kaixin Yang, Shengyi Li, Yanliang Sun, Andrew D. Cartmill, Ignacio F. López, Chunhui Ma, Qianbing Zhang

**Affiliations:** ^1^College of Animal Science and Technology, Shihezi University, Shihezi, Xinjiang, China; ^2^School of Agriculture and Environment, Massey University, Palmerston North, New Zealand

**Keywords:** phosphorus effectiveness, soil physical and chemical properties, soil phosphorus fractions, sustainable production, alfalfa (*Medicaco sativa* L.)

## Abstract

Nitrogen (N) and phosphorus (P) fertilizers change the morphological structure and effectiveness of P in the soil, which in turn affects crop growth, yield, and quality. However, the effects and mechanism of combined N and P application on the content of P fractions and the transformation of effective forms in alfalfa (*Medicago sativa* L.) production is unclear. This experiment was conducted with four levels of N: 0 (N_0_), 60 (N_1_), 120 (N_2_) and 180 kg·ha^-1^ (N_3_); and two levels of P (P_2_O_5_): 0 (P_0_) and 100 kg·ha^-1^ (P_1_). The results indicated that, under the same N level, P application significantly increased soil total N, and total P, available P, and content of various forms of inorganic P when compared to no P application, while decreasing the content of various forms of organic P and pH value. In general, under P_0_ conditions, soil total N content tended to increase with increasing N application, while total P, available P content, pH, inorganic P content in all forms, and organic P content in all forms showed a decreasing trend. When compared to no N application, insoluble P (Fe-P, O-P, Ca_10_-P) of the N application treatments was reduced 2.80 - 22.72, 2.96 - 20.42, and 5.54 - 20.11%, respectively. Under P_1_ conditions, soil total N and O-P tended to increase with increasing N application, while, pH, Ca_2_-P, Al-P, Fe-P, Ca_10_-P, and organic P content of each form tended to decrease. Total P, available P, and labile organic P (LOP) of N application reduced 0.34 - 8.58, 4.76 - 19.38, and 6.27 - 14.93%, respectively, when compared to no application. Nitrogen fertilization reduced the soil Ca_2_-P ratio, while P fertilization reduced soil Fe-P, moderately resistant organic P (MROP), and highly resistant P (HROP) ratios, and combined N and P elevated the Ca_8_-P to LOP ratio. The results of redundancy analysis showed that soil total N content, available P content, and pH were the key factors affecting the conversion of P fractions in the soil. Nitrogen and P reduced the proportion of soil insoluble P, promoted the activation of soil organic P, resulting in accumulation of slow-acting P in the soil, thereby improving the efficiency of soil P in alfalfa production.

## 1 Introduction

Alfalfa (*Medicago sativa* L.) is a cool-season, short lived, high yielding, perennial legume, with a deep tap root, and is an important source of good quality, palatable feed for a variety of livestock production systems (Ren et al., 2021). Nitrogen (N) and phosphorus (P) are the primary limiting nutrients for alfalfa growth and development (Du et al., 2020; Hu et al., 2023). Alfalfa forms symbiotic (mutually beneficial) relationships with nitrogen (N) fixing bacteria (rhizobia) which reside in nodules on alfalfa roots. These rhizobia can convert atmospheric N into plant available forms. However, despite the N fixing capabilities of these rhizobacteria, alfalfa may experiences N deficiency, due to inadequate soil nutrients (Zielewicz et al., 2023) salt stress (Wan et al., 2023), drought (Liu et al., 2018), and other factors, which ultimately reduce plant growth, yield, and quality (Zhao et al., 2022).

The majority of P in the soil exists as insoluble and organic P, resulting in reduced soil P availability for plant growth (Mirriam et al., 2022; Li et al., 2022c). Phosphorus in plants is highly mobile and allocation of P in plants is an important strategy for improving N and P utilization efficiency under nutrient deficiency (Seleiman et al., 2020b). Consequently, in alfalfa production, it is common to apply additional N and P fertilizers to increase yield and enhance quality (Li et al., 2022a).

Application of P fertilizer can effectively increase the levels of total and available P in the soil (Li et al., 2022b). Additionally, application of N fertilizer can enhance root growth and P uptake (Elgharably and Benes, 2021). However, only a small portion of the applied P fertilizer is typically utilized by alfalfa. In that, a large portion of applied P fertilizer becomes fixed and accumulates in the soil profile, forming organic and insoluble inorganic P, which is difficult for alfalfa to directly absorb (Li et al., 2022a). Reduced soil P availability not only reduces alfalfa growth and quality, but also represents a significant economic and environmental challenge, relating to fertilizer and application costs, and surface and groundwater impairment (Gu et al., 2023).

The effectiveness of soil P refers to the extent to which P can be absorbed and utilized by plants, with different forms of P present in the soil as the primary reasons for variations in P effectiveness (Li et al., 2022c). Therefore, subdividing soil P based on its forms plays a crucial role in studying P supply status in the soil and assessing the risk of P loss. Inorganic P grading method, primarily used in northern China’s calcareous soils, divides inorganic P into various forms, for example NaHCO_3_-soluble P (Ca_2_-P type), NH_4_Ac-soluble P (Ca_8_-P type), NH_4_F (Al-P type), NaOH-CaCO_3_-soluble P (Fe-P type), closed-accumulation P (O-P type), and H_2_SO_4_-soluble P (Ca_10_-P type) (Gu and Jiang, 1990). Inorganic P is primarily dominated by Ca-P, out of which insoluble Ca_10_-P content is predominant. It has been shown that P application increases inorganic P content in dryland calcareous soils, and the effectiveness is shown as Ca_2_-P > Al-P > Ca_8_-P > Fe-P > O-P > Ca_10_-P (Xiong et al., 2021). In terms of organic P, the Bowman-Cole classification method is commonly used, which categorizes organic P into four classes: labile organic P (LOP), moderately labile organic P (MLOP), moderately resistant organic P (MROP), and highly resistant organic P (HROP) (Bowman and Cole, 1978). Among these, LOP and MLOP are more prone to mineralization and decomposition, serving as potential P sources for plant growth and promoting the utilization of soil P by plants. However, MROP and HROP have relatively stable structures and are generally not readily absorbed by plants (Boschetti et al., 2009). Research has revealed that the application of N fertilizer during the fertilization process alters the pH of the soil and affects the migration rate of P, thereby enhancing the plant’s ability to absorb P (Elgharably and Benes, 2021). Conversely, P application promotes the transformation of soil insoluble organic P into forms that are readily available for plant uptake (Zheng et al., 2023).

In the Xinjiang region of China, soils are predominantly alkaline, and have a strong capacity to fix P, which negatively affects crop production, as P is more likely to be adsorbed and transformed into insoluble inorganic forms. Thus, uncoupling the mechanisms of P fraction transformation is of significant importance for improving P utilization efficiency. Application of N and P fertilizers, and transformation, accumulation, and availability of P in the soil has garnered increasing interest (Guera and Adriel, 2022; Liu et al., 2023), as has classification, spatial distribution, and bioavailability of soil P (Zeng et al., 2022). However, most of the existing studies have been on single application of N or P fertilizers (Amin, 2023; Huang et al., 2023), and the impact of combined N and P fertilization on the forms of soil P and the underlying response mechanisms with soil physical and chemical properties remain unclear.

Therefore, the objective of this study was to understand the effects of different N and P fertigation treatments on P fraction transformation and availability in the soil for alfalfa production. Additionally, this study aims to explore the correlation between P forms and soil physicochemical properties. Through testing the hypothesis that increasing application rates of N and P fertilizers, will decrease both the content of insoluble inorganic P and stable organic P, while increasing the proportion of the active P in the soil, we will provide valuable insights for improving P utilization efficiency and P transformation in alfalfa production. Furthermore, increasing economic constraints and environmental concerns will necessitate a more cautious utilization of nutrients/fertilizers during alfalfa production. We suggest that, a better understanding of the impact of combined N and P fertilization on the soil’s physical and chemical properties, as well as enhancing P utilization efficiency, holds significant importance in promoting sustainable and resilient alfalfa production.

## 2 Materials and methods

### 2.1 Experimental site description

**Figure 1:**
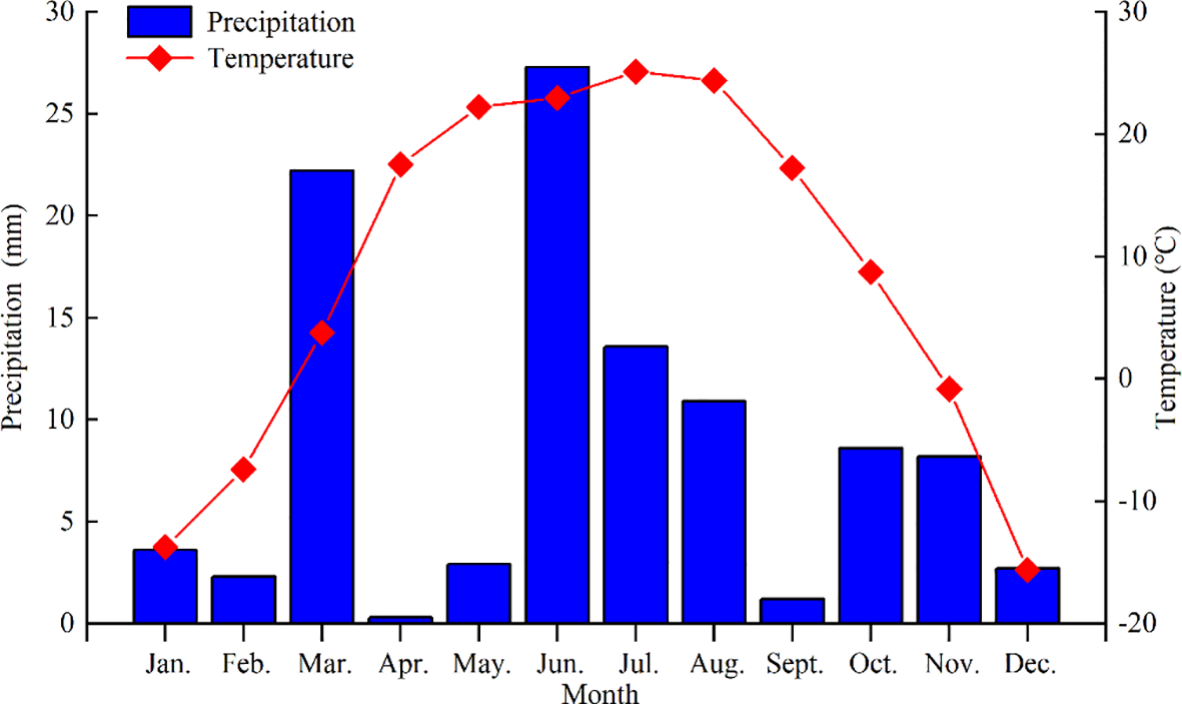
Monthly meteorological data during study period at the experiment site. Bars.

This experiment was conducted at the Water Conservation and Irrigation Laboratory at Shihezi University, Shihezi City, Xinjiang, China (44°20′ N, 88°30′ E). Arid climatic conditions were typical for the region, with cold winters (average temperature of -12.08 °C and minimum temperature of -30.90 °C) and hot summers (average temperature of 23.88°C and maximum temperature of 39.30 °C), annual rainfall of 110 - 200 mm, annual evaporation rate of 1000 - 1500 mm, and a 30 years average annual temperature of 7.9 °C. Rainfall and air temperature during the study period are summarized in **Figure 1**.

The soil type of the experiment site was grey desert soil, with basic physical and chemical properties, in the cultivated layer (0 - 20 cm), as follows: total N 1.18 g kg^−1^, total P 0.53 g kg^−1^, available P 19.30 mg·kg^−1^, available K 119.80 mg·kg^-1^, bulk density 1.49 g·cm^-3^, organic matter 21.56 g·kg^-1^, and soil pH 8.17.

### 2.2 Experimental design and management

The experiment was conducted as a two-factor randomized block design with four N application gradients: 0 (N_0_), 60 (N_1_), 120 (N_2_), and 180 (N_3_) kg·ha^-1^; and two P (P_2_O_5_) application gradients: 0 (P_0_), and 100 (P_1_) kg·ha^-1^, for a total of eight nitrogen and phosphorus treatments: N_0_P_0_, N_1_P_0_, N_2_P_0_, N_3_P_0_, N_0_P_1_, N_1_P_1_, N_2_P_1_, N_3_P_1_, with three replications per treatment.

Grass plots 24 m^2^ (4 m × 6 m) were established in April 2019, with a 1 m wide isolation strip between the plots to prevent water and nutrient movement between the plots. Plots were deep-tilled and cleared of debris prior to planting. Seed of alfalfa WL366HQ, an adaptable, high yielding and quality variety, was manually strip-seeded at a rate of 18 kg·ha^-1^, with planting rows spaced 20 cm apart, at a depth of 2 cm. Inlaid drip irrigation tapes were buried 10 -15 cm below the soil surface and spaced 60 cm apart, with drip heads spaced 20 cm apart.

Urea (46% N) and monoammonium P (52% P, 12.2% N) were used as the N and P source, respectively. In order to ensure that the test was only affected by the P fertilizer, the effect of N in the monoammonium P was counteracted by applying urea. Fertilizers were added to the fertigation tanks, and was drip applied with water 3–5 d after cutting (first flowering stage) on 9 May, 30 May, 6 July, and 12 August 2020.

### 2.3 Collection of soil samples

After each crop of alfalfa was cut, a five-point (“S” pattern) soil sampling method was used, l samples (0–20 cm) were taken using a soil auger (5 cm in diameter), composited, mixed thoroughly, and then passed through a coarse sieve (2 mm) to remove roots and debris. Soil samples were processed using the quadratic method and duplicate samples were retained; with one sample air-dried for the determination of soil physicochemical properties and P fraction content, and the remaining sample was stored in a refrigerator (-20°C) for reference.

### 2.4 Determination of physical and chemical properties of soil

Total N content was determined by Kjeldahl method (Lu, 2000). Total P content was determined by sulfuric acid-perchloric acid decoction method (Lu, 2000). Available P content was extracted with NaHCO_3_ and determined using the molybdenum antimony colorimetric method (Lu, 2000). Soil pH was measured using a portable pH meter, with a soil-to-water ratio of 2.5:1. (LEICI, INC, Shanghai, China).

### 2.5 Determination of soil phosphorus fractions

Inorganic P fractions were determined by the Sequential fractionation method (SFRM) (Gu and Jiang, 1990), which extracts inorganic P in the order of Ca_2_-P, Ca_8_-P, Al-P, Fe-P, O-P, and Ca_10_-P. Organic P fractions were determined by the Bowman-Cole method (Bowman and Cole, 1978), which categorizes organic P into LOP, MLOP, MROP and HROP. The extraction reagents were shown in **Table 1**.

**Table 1:** Extraction reagent of phosphorus fraction.

Sequence	Inorganic P Fractions	Extractants	Shaking time
First	Ca_2_-P	0.25M NaHCO_3_	1h
Second	Ca_8_-P	0.5M CH_3_COONH_4_	4h stand, 1h
Third	Al-P	1M NH_4_Cl, 0.5M NH_4_F	0.5h, 1h
Fourth	Fe-P	0.1M NaOH-Na_2_CO_3_	2 h, 16 h stand, 2 h
Fifth	O-P	0.3M Na_3_C_6_H_5_O_7_, 0.5M NaOH	15min,10min,1h
Sixth	Ca_10_-P	0.25M H_2_S0_4_	1h
	Organic P fractions		
First	LOP	0.5M NaHCO_3_	3h stand
Second	MLOP	1.0M H_2_S0_4_ + 0.5M NaOH	6h stand
Third	MROP	0.5M NaOH (no precipitation at pH1~1.8)	12h stand
Fourth	HROP	0.5M NaOH (precipitate at pH1~1.8)	

### 2.6 Data processing

Data was compiled using Microsoft Excel 2016 (Microsoft Corp., Redmond, WA, USA), SPSS 22.0 (SPSS Inc., Chicago, IL, USA) was used for ANOVA, and Duncan’s method was used for significance of difference analysis (α = 0.05). Redundancy analysis and Pearson correlation analysis of date were conducted using Canoco 5.0 (Microcomputer Power, Ithaca, NY, USA) and Origin 2019b (OriginLab Corp., Northampton, MA, USA), respectively.

## 3 Results

### 3.1 Soil nutrient content and pH value

Both N and P single fertilization had significant (*p* < 0.05) effects on alfalfa total soil N, total P, available P content, and pH. Combined N and P had significant (*p* < 0.05) effects (on soil total P and available P content (except for the first crop available P content) (**Table 2**).

**Table 2:** Soil physical and chemical properties of alfalfa under different nitrogen (N) and phosphorus (P) fertigation.

Treatment	First cut		Second cut		Third cut		Fourth cut	
	Total N (g·kg^-1^)	Total P (g·kg^-1^)	Total N (g·kg^-1^)	Total P (g·kg^-1^)	Total N (g·kg^-1^)	Total P (g·kg^-1^)	Total N (g·kg^-1^)	Total P (g·kg^-1^)
N_0_P_0_	0.72 ± 0.015Bd	0.48 ± 0.004Ba	0.75 ± 0.015Bd	0.45 ± 0.004Ba	0.71 ± 0.015Bd	0.42 ± 0.004Ba	0.70 ± 0.015Bd	0.47 ± 0.004Ba
N_1_P_0_	0.91 ± 0.019Bc	0.45 ± 0.003Bb	0.88 ± 0.016Bc	0.43 ± 0.003Bb	0.94 ± 0.040Bc	0.42 ± 0.003Ba	1.03 ± 0.026Bc	0.41 ± 0.007Bb
N_2_P_0_	0.96 ± 0.023Bb	0.43 ± 0.002Bc	0.97 ± 0.020Bb	0.42 ± 0.002Bc	1.06 ± 0.016Bb	0.40 ± 0.002Bb	1.11 ± 0.026Bb	0.41 ± 0.005Bb
N_3_P_0_	1.12 ± 0.021Ba	0.42 ± 0.002Bd	1.15 ± 0.018Ba	0.39 ± 0.002Bd	1.25 ± 0.019Ba	0.39 ± 0.002Bc	1.33 ± 0.025Ba	0.38 ± 0.005Bc
N_0_P_1_	0.86 ± 0.010Ad	0.52 ± 0.006Aa	0.81 ± 0.010Ad	0.59 ± 0.007Aa	0.83 ± 0.009Ad	0.61 ± 0.010Aa	0.78 ± 0.009Ad	0.71 ± 0.013Aa
N_1_P_1_	1.06 ± 0.008Ab	0.50 ± 0.002Ab	0.96 ± 0.011Ac	0.58 ± 0.003Ab	1.06 ± 0.010Ac	0.59 ± 0.004Ab	1.09 ± 0.020Ac	0.68 ± 0.005Ab
N_2_P_1_	1.03 ± 0.015Ac	0.49 ± 0.006Ac	1.15 ± 0.005Ab	0.55 ± 0.005Ac	1.20 ± 0.012Ab	0.59 ± 0.011Ab	1.15 ± 0.010Ab	0.67 ± 0.002Abc
N_3_P_1_	1.20 ± 0.018Aa	0.48 ± 0.002Ad	1.24 ± 0.025Aa	0.56 ± 0.003Ac	1.43 ± 0.023Aa	0.61 ± 0.004Aa	1.37 ± 0.028Aa	0.67 ± 0.005Ac
F_N_	310.64^**^	1390.30^**^	272.04^**^	6163.48^**^	319.36^**^	6226.10^**^	73.39^**^	7902.83^**^
F_P_	611.00^**^	248.82^**^	851.68^**^	125.02^**^	947.39^**^	14.12^**^	1604.65^**^	90.26^**^
F_N_×F_P_	9.68^**^	5.02^*^	17.03^**^	8.56^**^	2.80^ns^	17.85^**^	1.96^ns^	6.58^**^
Treatment	First cut		Second cut		Third cut		Fourth cut	
	Available P (mg·kg^-1^)	pH value	Available P (mg·kg^-1^)	pH value	Available P (mg·kg^-1^)	pH value	Available P (mg·kg^-1^)	pH value
N_0_P_0_	7.99 ± 0.113Ba	8.18 ± 0.02Aa	7.76 ± 0.113Ba	8.17 ± 0.01Aa	7.21 ± 0.113Ba	8.16 ± 0.01Aa	7.35 ± 0.113Ba	7.79 ± 0.01Aa
N_1_P_0_	7.41 ± 0.267Bb	8.13 ± 0.01Ab	7.58 ± 0.192Ba	8.10 ± 0.01Ab	6.53 ± 0.209Bb	8.08 ± 0.03Ab	6.07 ± 0.140Bb	7.73 ± 0.02Ab
N_2_P_0_	6.84 ± 0.301Bc	8.12 ± 0.01Ab	6.21 ± 0.166Bb	8.09 ± 0.02Abc	5.83 ± 0.104Bc	8.01 ± 0.02Ac	5.75 ± 0.095Bc	7.70 ± 0.01Ac
N_3_P_0_	6.29 ± 0.252Bd	8.06 ± 0.00Ac	6.03 ± 0.145Bb	8.06 ± 0.02Ac	5.48 ± 0.098Bd	7.98 ± 0.03Ac	5.25 ± 0.202Bd	7.57 ± 0.01Ad
N_0_P_1_	10.76 ± 0.160Aa	8.16 ± 0.01Ba	13.26 ± 0.080Aa	8.14 ± 0.01Ba	15.88 ± 0.269Aa	8.13 ± 0.03Aa	18.56 ± 0.371Aa	7.66 ± 0.01Ba
N_1_P_1_	10.25 ± 0.341Ab	8.10 ± 0.02Bb	12.57 ± 0.253Ab	8.07 ± 0.00Bb	13.75 ± 0.307Ab	8.04 ± 0.03Bb	16.93 ± 0.455Ab	7.56 ± 0.02Bb
N_2_P_1_	9.80 ± 0.196Ac	8.10 ± 0.01Ab	11.73 ± 0.225Ac	8.06 ± 0.01Bb	12.84 ± 0.330Ac	8.02 ± 0.02Ab	16.34 ± 0.235Ab	7.53 ± 0.01Bb
N_3_P_1_	9.59 ± 0.116Ac	8.04 ± 0.01Bc	11.04 ± 0.162Ad	8.02 ± 0.01Bc	12.80 ± 0.156Ac	7.89 ± 0.02Bc	15.37 ± 0.342Ac	7.40 ± 0.01Bc
F_N_	865.88^**^	62.49^**^	4935.57^**^	47.22^**^	6746.25^**^	3.93^ns^	9413.65^**^	256.31^**^
F_P_	38.46^**^	288.99^**^	152.36^**^	109.36^**^	141.39^**^	18.54^*^	101.68^**^	96.89^*^
F_N_×F_P_	1.36^ns^	0.12^ns^	3.85^*^	0.71^ns^	16.78^**^	4.17^*^	4.39^*^	2.06^ns^

Soil samples were taken on May 22 (first cut), June 26 (second cut), August 12 (third cut) and October 4 (fourth cut), 2020, respectively. Different capital letters indicated that there were significant differences among different phosphorus treatments under the same nitrogen application level (*p* < 0.05). Different lowercase letters indicated significant difference in nitrogen application under the same phosphorus application level (*p* < 0.05). ** means very significant difference (*p* < 0.01), * means significant difference (p < 0.05), ns means no significant difference (*p* > 0.05).

In general, under the same N treatment, with increasing P fertilizer application, soil total N, total P, and available P content increased. P_1_ was increased total N, total P and available P content by 3.25 - 18.55, 8.92 - 74.79, 34.67 - 192.71 and 0.24 - 1.34%, respectively, when compared to P_0_. In general, under the same P fertilizer treatments, with increased N application, total soil N increased gradually (except for the 1st crop of P_1_, which increased initially and then decreased) and reached a maximum value under the N_3_ treatment, and was significantly (*p* < 0.05) higher than the rest of the N application treatments. Total soil P and available soil P content and soil pH showed a gradual decreasing trend (except for the 3rd crop of P_2_, which had an increasing - decreasing - raising trend). Nitrogen application under P_0_ conditions elevated soil total N by 17.22 - 89.63%, and decreased soil total P, available P content, and pH by 0.28 - 18.45, 2.24 - 28.51 and 0.73 - 2.98%, respectively. Nitrogen application under P_1_ conditions elevated soil total N content by 17.71 - 76.51%, and soil total P, available P content, while pH decreased by 0.34 - 8.58, 4.76 - 19.38 and 0.65 - 3.29%, respectively.

### 3.2 Soil total inorganic phosphorus and total organic phosphorus content

**Figure 2:**
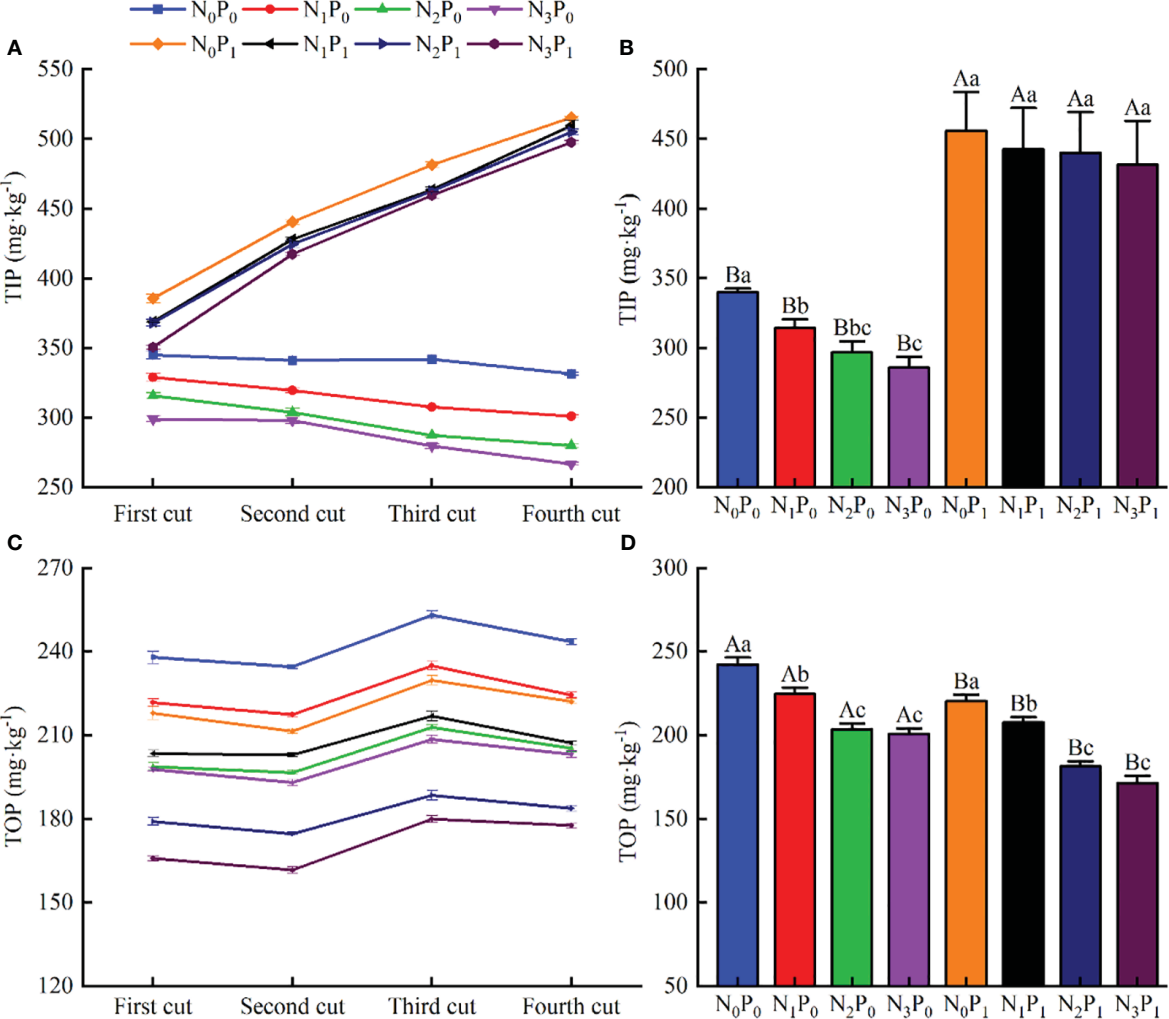
Total inorganic phosphorus (TIP; mg kg^-1^) and total organic phosphorus (TOP; mg kg^-1^) content of soil under alfalfa production with different fertilization treatments; **(A)** TIP content, **(B)** mean annual TIP content of each treatment, **(C)** TOP content, and **(D)** mean annual TOP of each treatment (mean +- SE). Different capital letters indicate significant (*p* < 0.05) differences among treatments with different phosphorus (P) applications at the same nitrogen (N) application level; different lowercase letters indicate significant (*p* < 0.05) differences among treatments with different N applications at the same P application level.

Nitrogen and P single fertilization and N-P combined had highly significant (*p* < 0.01) effects on soil total inorganic P and total organic P content (**Figures 2A, C**). Under the same N application treatment, soil total inorganic P content showed an upward trend with increasing P application. P_1_ increased by 11.82 - 86.50%, when compared with P_0_ (**Figure 2A**). Total organic P content decreased, and P_1_ decreased by 4.77 - 24.97%, respectively, when compared with P_0_ (**Figure 2A**). Under the same P treatment, increasing N fertility resulted in decreased total soil inorganic P and total organic P contents of alfalfa showed a gradual decreasing trend and reached the lowest value under N_3_ treatment (**Figures 2B, D**). Nitrogen application treatments were all significantly (*p* < 0.05) smaller than the no N application treatments (except for the total organic P content at the level of P_1_ in the 2nd crop). Total inorganic P and total organic P contents of alfalfa were reduced by 4.66 - 19.51 and 6.90 - 17.58%, respectively in N application treatments when compared with no N application treatments under P_0_ conditions, and 1.11 - 9.05 and 6.64 - 28.20% under P_1_ conditions (*p* < 0.05) (**Figures 2A, C**).

### 3.3 Content of soil inorganic phosphorus fraction

**Figure 3:**
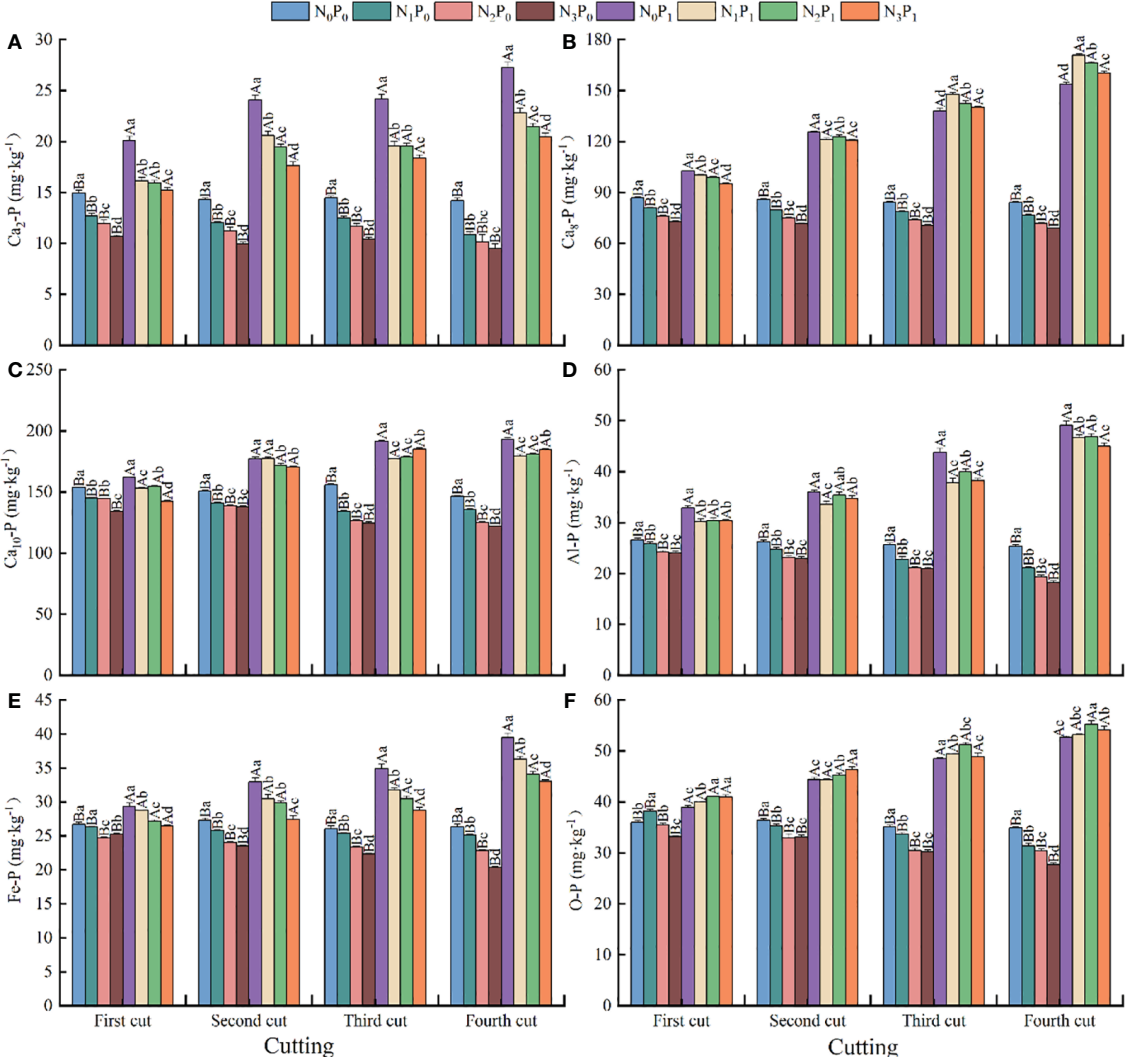
Soil inorganic phosphorus (P) fractions, **(A)** Ca_2_-P, **(B)** Ca_8_-P, **(C)** Ca_10_-P, **(D)** Al-P, **(E)** Fe-P, and **(F)** O-P contents, respectively, in alfalfa production under different fertilization treatments. (mean +- SE). Different capital letters indicate significant (*p* < 0.05) differences among treatments with different P applications at the same nitrogen (N) application level; different lowercase letters indicate significant (*p* < 0.05) differences among treatments with different N applications at the same P application level.

Nitrogen application, P application, and N-P rationing all had significant (*p* < 0.05) effects on the content of inorganic P fractions in soils under alfalfa production (**Figure 3**). Under the same N application conditions, with increased P application, the Ca_2_-P, Ca_8_-P, Al-P, Fe-P, O-P, Ca_10_-P contents of soil under alfalfa production showed an upward trend. Phosphorus application increased when compared with no applied P by 27.06 - 114.89, 17.92 - 132.90, 16.76 - 146.09, 4.44 - 62.61, 4.77 - 94.95, and 5.3 - 51.23%, respectively, and the upward trend increased with the increase of cutting stubble. Under P_0_ conditions, the Ca_2_-P, Ca_8_-P, Al-P, Fe-P, O-P, and Ca_10_-P contents of soils showed a gradual decreasing trend (except for the first crop where Fe-P showed a decreasing and then increasing trend, and the first and third crops where O-P showed an increasing and then decreasing trend) with the increase in N application, and reached the minimum value under N_3_ treatment. Nitrogen application reduced 13.73 - 33.01, 6.67 - 18.07, 2.98 - 28.02, 2.80 - 22.72, 2.96 - 20.42, and 5.54 - 20.11%, respectively, when compared to no application. Under P_1_ conditions, soil Ca_2_-P and Fe-P showed a gradual decreasing trend with increased N application and reached the minimum value under N_3_ treatment, and N application reduced 14.42 - 26.79 and 2.07 - 17.64%, respectively, when compared with no applied N treatment. Soil Ca_8_-P, Al-P, and Ca_10_-P in the N treatment was significantly (*p* < 0.05) smaller than that of the unapplied N treatment [except for the third and Ca_8_-P in the 4th crop was significantly (*p* < 0.05) larger than that in the unapplied N treatment; the Ca_10_-P in the 2nd crop under N_1_ treatment and Al-P under N_2_ treatment were not significant; the O-P showed a trend of increasing and then decreasing except for the 2nd crop, which showed a trend of decreasing and then increasing], and reached the maximum value under N_2_ treatment.

### 3.4 Content of each organic phosphorus fraction of soil

**Figure 4:**
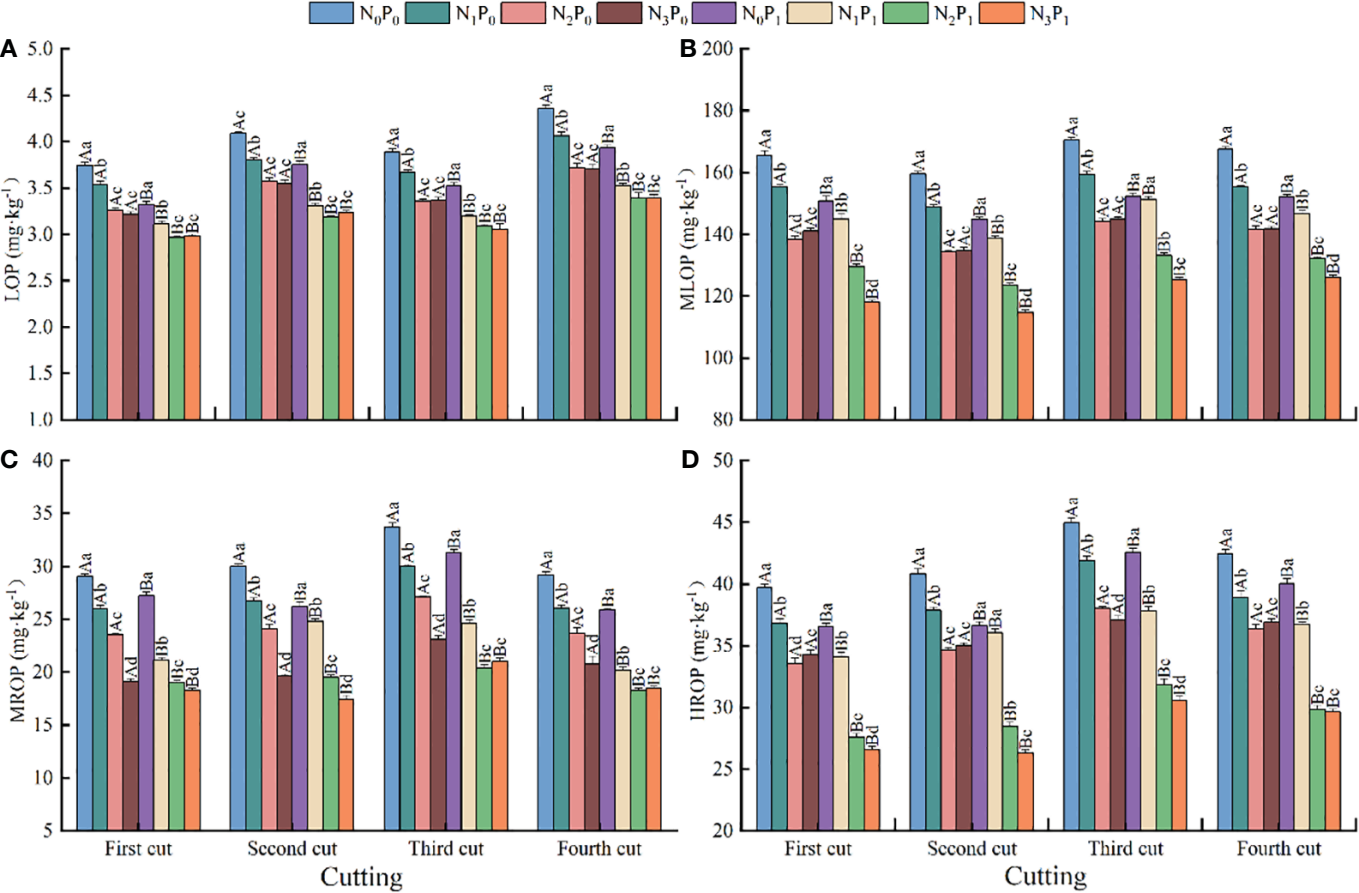
Content of organic phosphorus (P) fractions, **(A)** LOP, **(B)** MLOP, **(C)** MROP, and **(D)** HROP soil under different treatments (mean +- SE). Different capital letters indicate significant (*p* < 0.05) differences among treatments with different P applications at the same nitrogen (N) application level; different lowercase letters indicate significant (*p* < 0.05) differences among treatments with different N applications at the same phosphorus application level.

The effects of N application, P application, and N-P combined the content of organic P fractions in soils under alfalfa production were highly significant (*p* < 0.01) (**Figure 4**). Under the same N application condition, with the increase of P application, LOP, MLOP, MROP, and HROP in soil showed a significant (*p* < 0.05) decreasing trend, and P application reduced 7.27 - 13.27, 5.13 - 16.35, 4.49 - 24.90 and 4.77 - 24.97% when compared with the no P application, respectively. Under P_0_ conditions, with increased N application, the contents of LOP, MLOP and MROP in alfalfa soil showed a decreasing trend (except for the 1st crop where the MLOP content showed a decreasing and then increasing trend), and the N application treatments were significantly (*p* < 0.05) reduced by 5.44 - 14.99, 6.15 - 16.37 and 10.32 - 34.46%, respectively, when compared with that of the unapplied N treatment. The HROP content of soil under alfalfa production, except for the 3rd crop which showed a gradual decreasing trend and then increasing trend (reaching the minimum value under N_2_ conditions), and its content of N application treatments was significantly (*p* < *0.05*), smaller than that of the unapplied treatments, by 6.90 - 17.58%. The LOP, MLOP, and HROP contents of soils under alfalfa production showed a significant (*p* < *0.05*) decreasing trend under P_1_ conditions (LOP contents did not differ significantly between N_2_ and N_3_ treatments), and the N application treatments were reduced by 6.27 - 14.93, 3.56 - 21.74 and 6.64 - 28.20%, respectively, when compared with no applied N treatments (*p* < 0.05). The MROP content of alfalfa soil showed a gradual decrease with the increase of N application in the 1st and 2nd crops. In that, it showed a trend of decreasing and then increasing in the 3rd and 4th crops (reaching the minimum under N_2_ treatment), but the differences between N_2_ and N_3_ treatments were not significant.

### 3.5 Ratio of soil phosphorus fractions to total inorganic and organic phosphorus content

**Figure 5:**
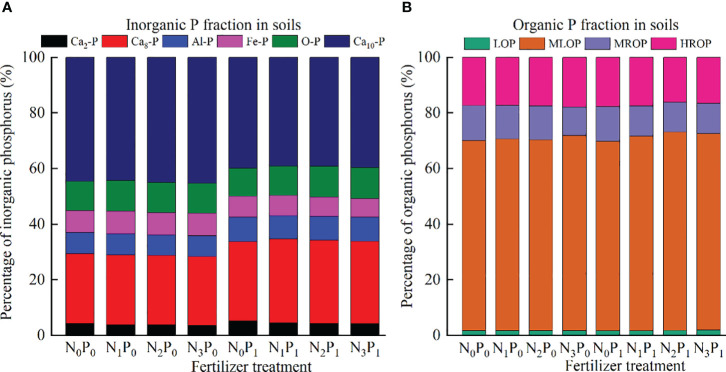
Percentage of each phosphorus (P) fraction of **(A)** inorganic P, and **(B)** organic P under different fertilization treatments.

All N and P treatments were dominated by Ca_10_-P for inorganic P and MROP for organic P (about 60% of the total) (**Figure 5**). Under the same N application conditions, P application increased the Ca_2_-P, Ca_8_-P, Al-P, and LOP proportions, decreased the Fe-P, MROP, and HROP proportions, and had a nonsignificant effect on the O-P and MLOP proportions (change of less than 5%), when compared with the no P application treatment. Under P_0_ conditions, the Ca_2_-P and MROP ratios tended to decrease with increasing N application and reached a minimum under N_3_ treatment, while the rest of the P form ratios did not change significantly. Under P_1_ conditions, the percentages of Ca_2_-P, Fe-P, MROP, and HROP showed a decreasing trend with increasing N application, all of which reached the minimum value under N_3_ conditions, the percentages of Ca_8_-P, O-P, and LOP showed an increasing trend, and the percentages of Al-P, Ca_10_-P, and MLOP showed insignificant changes.

### 3.6 Relationship between soil physicochemical properties and content of inorganic and organic phosphorus fractions

**Figure 6:**
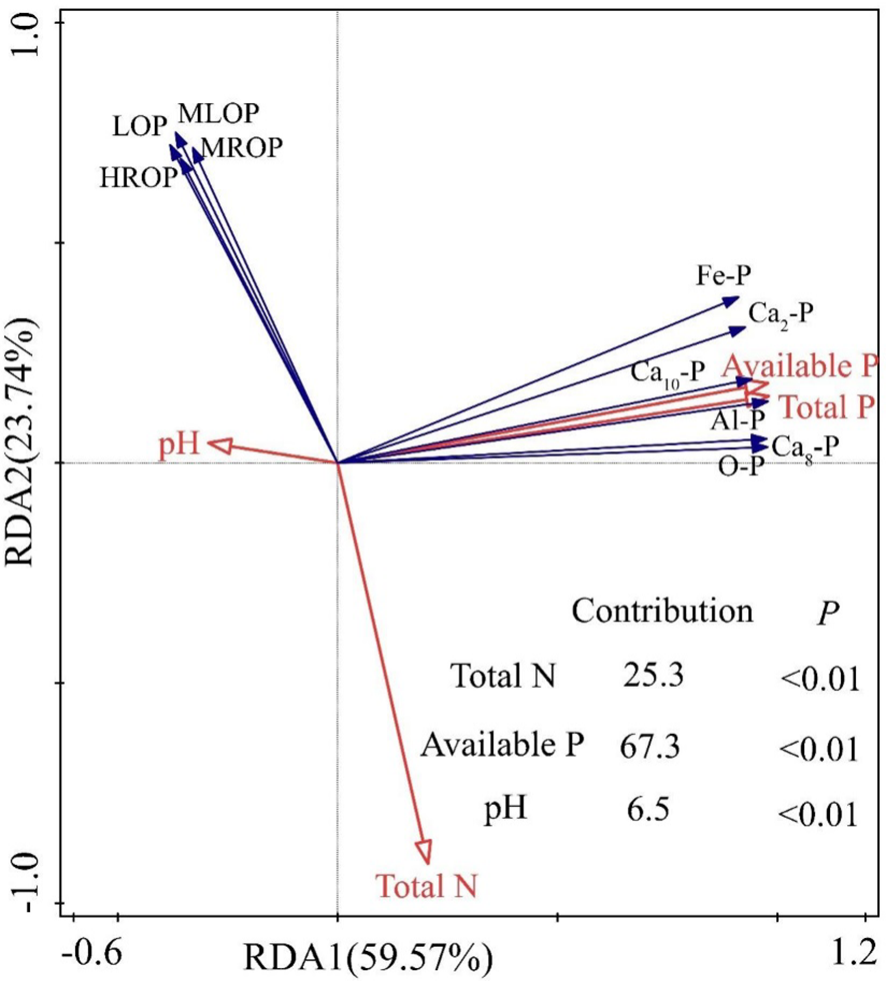
Relationship between soil physical and chemical properties and phosphorus (P) fractions under different fertilization treatments.

**Figure 7:**
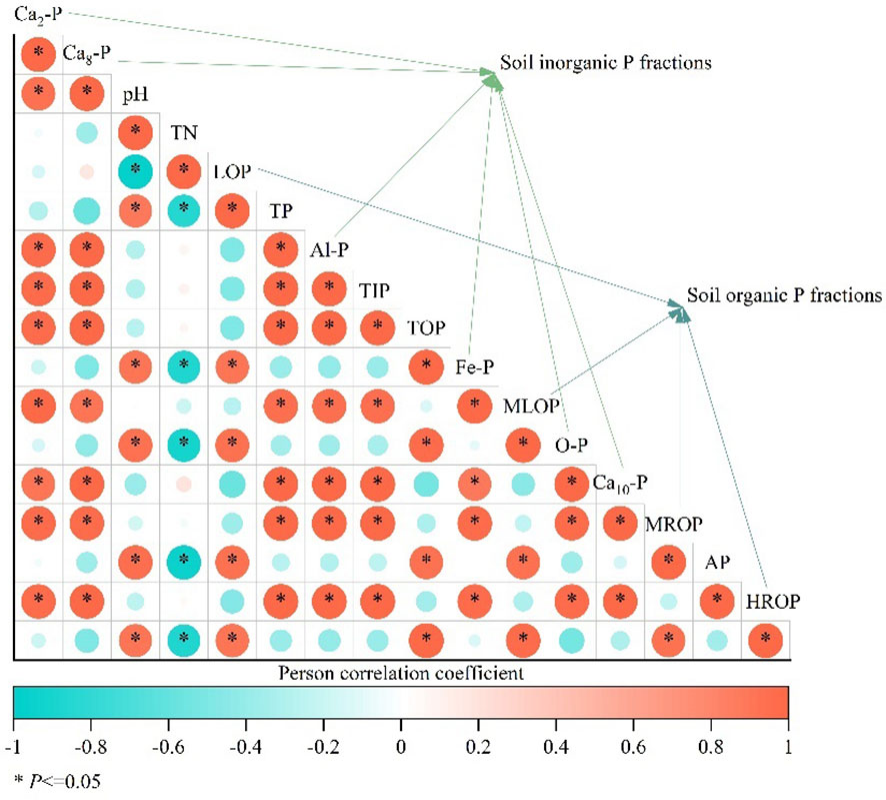
Pearson correlation analysis of soil physical and chemical properties and inorganic phosphorus (P) and organic P fractions. * indicates significant difference (*p* < 0.05).

Soil available P, total N, and pH were the main drivers affecting soil P fractions, explaining 67.3%, 25.3%, and 6.5%, of their variation, respectively (**Figure 6**). For the content of P fractions in soil (**Figure 7**), soil inorganic P fractions were significantly (*p* < 0.05) positively correlated with each other, and all of them were significantly (*p* < 0.05) positively correlated with soil total P, total inorganic P, and available P. Soil organic P fractions were significantly (*p* < 0.05) positively correlated with each other, all of them were highly positively correlated with soil total organic P (*p* < 0.05), and significantly (*p* < 0.05) negatively correlated with soil total N. pH and Total N were significantly (*p* < 0.05) negatively correlated with each other.

## 4 Discussion

### 4.1 Effects of nitrogen and phosphorus rationing on physicochemical properties of soil under alfalfa production

Soils are the main source of nutrients required for crop growth and N and P content, and thus their physicochemical properties are important for crop nutrient utilization (Seleiman et al., 2017, 2020a). Previous study have demonstrated that the application of N and P fertilizers effectively increases the levels of N and P in the soil during the growing season for alfalfa (Liu et al., 2022). In our study, the application of N and P fertilizers increased the total N content, while the application of P fertilizers significantly increased the total and available P content. At the same level of P fertilizer, total P and available P content of soil under alfalfa production showed a gradual decrease with increased N fertilization (**Table 2**). This increase indicates that N and P fertilizers are stored in the soil in a slow-acting form, while P fertilizer application promotes the development of alfalfa rhizobacteria (Qu et al., 2020), which in turn increases the amount of immobilized N in the soil. Nitrogen sufficiency increased alfalfa growth and root development, which in turn increasing soil P demand and uptake (Song et al., 2022). Increased N application, resulted in a smaller decrease in total and available P content under P fertilizer treatment when compared to no P application. This indicates that the regulation of N fertilizer on P uptake capacity of alfalfa may be related to the content of P in the soil. When soil P is deficient, the effect of N fertilizer in promoting P uptake by alfalfa is more pronounced (Tong et al., 2023).

Soil pH is directly related to the utilization of nutrients by the crop, and either too acidic or alkaline can result in the inability of the crop root system to absorb nutrients (Ringrose and Neilsen, 2005). Previous studies have shown that N application decreases soil pH, while P application has no significant effect on soil pH (An et al., 2022). Our study indicates that both N and P application have different abilities in lowering soil pH (**Table 2**). This could be attributed to the increased activity of rhizosphere microorganisms in alfalfa roots stimulated by N, resulting in more secretion from alfalfa roots and subsequently increasing soil acidity (An et al., 2022), and/or P fertilizer may lower pH by releasing H^+^ ions and promoting the binding of P ions with metal ions (Liu et al., 2022).

### 4.2 Effects of nitrogen and phosphorus rationing on inorganic phosphorus fractions in soils under alfalfa production

Inorganic P is the main source of P in the soil, and changes in its content can be used as an important assessment of P uptake by plants (Urlić et al., 2023). It has been shown that N application promotes the utilization of soil P by the plant and that an appropriate ratio of N to P helps to increase soil P content (Sun et al., 2022). In our study, soil TIP content showed a decreasing trend with increasing N application (**Figure 2A**). This trend may reflect enhanced photosynthesis and accelerated root growth in alfalfa from N fertilizer application, which in turn reduced the P content of the soil (Lu et al., 2019).

Soil reactive P Ca_2_-P and Ca_8_-P content was reported to decrease with increasing N application, while the proportion of Ca_2_-P to inorganic P increased (Cui et al., 2019). In our study, when N fertilizer was applied alone, the content of each soil inorganic P fraction tended to decrease with increased N fertilizer application, while the proportion of Ca_2_-P to inorganic P decreased. However, the proportion of Ca_8_-P to inorganic P tended to increase with increasing N application under combined N and P conditions (**Figure 5A**). This indicates that the addition of N fertilizer not only promotes plant uptake of reactive P, but also facilitates the conversion and degradation of insoluble P to other P forms. Combined N and P application, optimizes soil quality conditions, provides a suitable environment for microbial growth, increases phosphatase activity, and ultimately facilitates the conversion of soil insoluble P to moderately stabilized forms of P (Cui et al., 2019; Wu and Yu, 2023). Differences between Ca_10_-P and O-P contents of P application and no P application treatment were widened with increasing cutting stubbles and were higher in the P application treatment than in the no P application treatment (**Figures 3C, F**). This indicates that P fertilizer accumulates residually in the soil, while insoluble P remains the dominant form of P storage and the main direction of P transformation in the soil (Urlić et al., 2023).

### 4.3 Effect of nitrogen and phosphorus rationing on organic phosphorus fractions of soils under alfalfa production

Although soil organic P is one of the main fractions of the soil P pool, it is difficult for plants to utilized directly, leading to a serious underutilization of P resources. Therefore, fraction breakdown of organic P and research on methods to improve its utilization efficiency are key steps towards efficient utilization of soil P reservoirs (Sulieman and Mühling, 2021). It has been demonstrated that the application of organic acids promotes the mineralization of insoluble organic P and reduces the content of stable organic P fractions (Wang et al., 2023). The results of our study demonstrate that both N and P fertilizer application could reduce the proportion of MROP to organic P in the soil (**Figure 5B**). This indicates that the effect of N and P fertilizers on organic P in soil in alfalfa production is mainly in the mineralization and degradation of medium stable organic P. Application of P fertilizer alone and N-P combined increase organic P in LOP in addition to decreasing the percentage of stable organic P. This may be related to the interaction between N and P fertilizers. Phosphorus fertilizer decreases soil pH, providing a suitable acid-base environment for organic P mineralizing microorganisms and enzymes (Timofeeva et al., 2022). Nitrogen fertilizer promotes the growth of alfalfa roots and enhances the absorption of P by rhizobium, leading to increased production and activity of alkaline phosphatase and other mineralization factors. These factors, in turn, promote the activation of stable organic P in the soil, converting it into forms of P that can be readily absorbed by plants (Koester et al., 2021).

### 4.4 Factors influencing the morphological transformation of alfalfa soil phosphorus fractions

The physicochemical properties of the soil have an important effect on the transformation of P fractions (Zhang et al., 2021; Hei et al., 2023), and combined N and P fertilizers increases N content, lowers soil pH, and replenishes the soil’s available P content. Previous study indicates that soil organic matter content and total N content are important factors influencing the chemical forms of P in soil (Hei et al., 2023). The results of this study demonstrate that available P content, total N content, and pH were the main factors driving the conversion of P fractions in soils under alfalfa production (**Figure 6**). This could be due to the close correlation between the level of available P and alfalfa root growth, which in turn affects the regulation of the plant’s conversion of soil P components. Soil pH is an important environmental condition for maintaining the vitality of root microbiota and insoluble P mineralization enzymes, thus influencing the transformation of soil P (Cui et al., 2019).

Nitrogen in the soil is an essential element for plant development and microbial activity. In addition to enhancing microbial vitality, it also reduces the adsorption capacity of minerals, including Al and Fe on P, thereby decreasing the proportion of insoluble P in the soil and increasing the effectiveness of soil P for plants (Zhang et al., 2022).

## 5 Conclusion

Combined application of N and P has less soil P residue than that of P fertilizer alone. Addition of N fertilizer facilitated the conversion and degradation of insoluble P to other P forms, which in turn promotes active plant uptake of P. Under P_0_ conditions, the Fe-P, O-P, and Ca_10_-P contents of soils showed a gradual decreasing trend with the increase in N application. Nitrogen application reduced 2.80 - 22.72, 2.96 - 20.42, and 5.54 - 20.11%, respectively, when compared to no N application. Under P_1_ conditions, the percentages of MROP and HROP showed a decreasing trend with increasing N application, while the percentages of Ca_8_-P, O-P, and LOP showed an increasing trend. Soil available P content, total N content and pH are closely related to the transformation of soil P fractions, therefore, the impact on soil physicochemical properties should be considered comprehensively when applying N and P fertilizers, in order to improve the efficiency of soil P use.

Our study provides a theoretical basis for the efficient use of N and P fertilizers in order to increase the content of soil active P fractions, which in turn provides a theoretical basis for minimizing farmers’ production costs, P loading and residues in the soil, and nutrient losses to the environment.

## Data availability statement

The original contributions presented in the study are included in the article/supplementary material, further inquiries can be directed to the corresponding author/s.

## Author contributions

KY: Writing – original draft, Validation, Formal Analysis, Data curation. SL: Writing – original draft, Conceptualization. YS: Writing – original draft, Validation, Software. IL: Writing – review & editing. AC: Writing – review & editing, Funding acquisition. CM: Writing – review & editing, Conceptualization. QZ: Writing – review & editing, Supervision, Funding acquisition, Data curation, Conceptualization.
